# Alveolar echinococcosis mimicking bilateral lung metastatic cancer

**DOI:** 10.1590/0037-8682-0140-2022

**Published:** 2022-08-05

**Authors:** Yener Aydin, Ali Bilal Ulas, Atilla Eroglu

**Affiliations:** 1 Ataturk University, Medical Faculty, Department of Thoracic Surgery, Erzurum, Turkey.

A 62-year-old male presented with dyspnea, cough, and severe chest pain complaints. The patient was diagnosed with hepatic alveolar echinococcosis 13 years ago. Widespread metastasis was seen in the posteroanterior chest radiograph of the patient, who was treated with albendazole (Figure 1).

**FİGURE 1:**
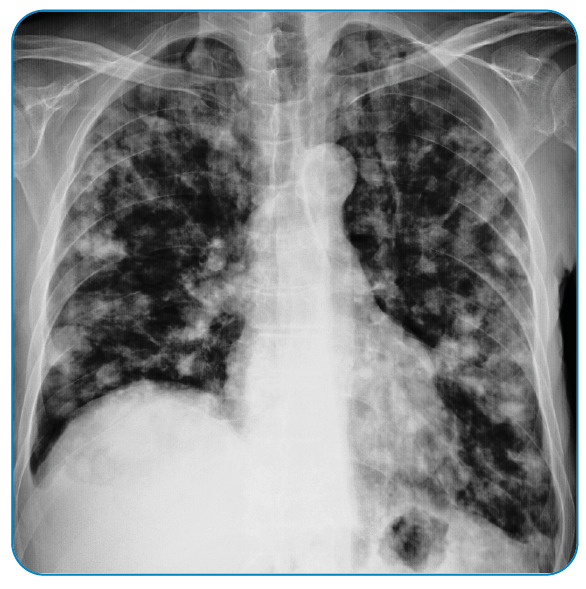
Chest radiograph shows multiple metastatic nodules in all lobes of both lungs.

The incidence of alveolar echinococcosis caused by the larval forms of the *Echinococcus multilocularis* has increased in recent years. Lung metastases occur in 7-20% of patients with liver involvement^1^ ^,^ ^2^. Alveolar echinococcosis behaves like a tumor. Treatment with albendazole may slow the growth of metastases^1^ in patients with diffuse metastatic nodules in the lung, and alveolar echinococcosis should be considered as a cause.
